# Socio-demographic correlates of betel, areca and smokeless tobacco use as a high risk behavior for head and neck cancers in a squatter settlement of Karachi, Pakistan

**DOI:** 10.1186/1747-597X-1-10

**Published:** 2006-04-26

**Authors:** Samia Mazahir, Rabia Malik, Maria Maqsood, Kanwal AliRaza Merchant, Farida Malik, Atif Majeed, Zafar Fatmi, Muhammad Rizwanulhaq Khawaja, Shehzad Ghaffar

**Affiliations:** 1 Class of 2006, Aga Khan University – Medical College, Karachi 74800, Pakistan; 2 Assistant Professor, Department of Community Health Sciences, Aga Khan University, Karachi 74800, Pakistan; 3 Senior Instructor, Department of Surgery (ENT, Head & Neck division), Aga Khan University, Karachi 74800, Pakistan

## Abstract

**Background:**

Head and neck cancers are a major cancer burden in Pakistan. They share a common risk factor profile including regular consumption of products of betel, areca and tobacco. Use of paan, chaalia, gutka, niswar and tumbaku is acceptable in Pakistan and is considered a normal cultural practice. This cross-sectional study was carried out to understand the relation of socio-demographic factors for the consumption of paan, chaalia, gutka, niswar and tumbaku in Pakistani population. Through systematic sampling, 425 subjects from a squatter settlement in Karachi were interviewed using a structured questionnaire. High risk behavior was defined as Daily use of any of the above products.

**Results:**

Daily use of all the substances except chaalia was higher among males compared to females. Chaalia use was higher among adolescents than adults while non-married consumed both chaalia and gutka more than married. Mohajir ethnicity had higher prevalence of paan, gutka and tumbaku use while Pathans had higher prevalence of niswar use.

**Conclusion:**

Prevalence of use of chewable products is high in Pakistan with particularly high use of certain substances related with socio-demographic profiles. Industrially prepared products, chaalia and gutka, are gaining popularity among youth. Policies and focused interventions can be developed taking into consideration the preferred use of products among different socio-demographic groups.

## Background

Areca nut, often used with betel quid and chewable tobacco is the fourth most commonly used psycho-active substance in the world, ranking after caffeine, alcohol and nicotine [1]. High prevalence of use of these items has been reported in South and South East Asia [1-3]. Having an ancient history, they are an integral part of the culture and sometimes erroneously believed to have medicinal benefits [4,5]. Population based surveys from India, Pakistan and Nepal over the past couple of decades have reported a prevalence of use of these products between 20–40% among adolescents and adults [1-3]. In Pakistan, a recent study among the adolescents and adults of a Karachi squatter settlement reported that 40% of the population was using at least one chewable product of betel, areca and tobacco on a daily basis [6].

Chewing betel, areca and smokeless tobacco products lead to discoloration of teeth, development of chronic debilitating diseases involving gingival and oral mucosa, and higher mortality among users. These diseases include oral submucous fibrosis, oral leukoplakias, oral cavity and other head and neck cancers [1,4,5,7,8]. Furthermore, a link between the use of betel and hepatocellular carcinoma has also been reported [8]. The incidence of head and neck cancers correlates with the geographical prevalence of these products as 58% of global head and neck cancers occur alone in South and Southeast Asia [9,10]. Time trend analysis has suggested a rise in the incidence of oral cavity cancers in Pakistan [11]. The incidence of oral cavity cancers in Karachi South district of Pakistan is the highest in the world [11].

In Pakistan and South Asian subcontinent, the popular chewing products are paan, chaalia, gutka, niswar and tumbaku [12]. The composition of these products may vary from one to another but the main ingredients remain betel, areca and tobacco. Paan contains areca nut, betel leaf and calcium hydroxide but tobacco and various other spices are also commonly added [5]. Industrially prepared mixture of areca nut, lime, catechin containing substance, sandalwood fragrance with tobacco (gutka) or without tobacco (chaalia) were introduced in recent decades, which have contributed to growth and use of these products. [1]. Tumbaku and niswar mainly contain tobacco with small amount of spices, areca and betel. Tumbaku is oral chewable form of tobacco while niswar is placed in oral vestibule [12].

Studies suggest that different socio-demographic groups prefer specific items. For instance, studies conducted in Karachi showed higher consumption of betel quid among boys than girls [13]. Differential chewing habits of paan and tumbaku among males and females have also been reported [3]. The differential use of these products across gender and region was also seen in India [1]. However, these studies were not specifically designed to look into the socio-demographic correlates of chewable products. Such endeavors can help develop policies to implement focused interventions. With these considerations in mind, we designed a study to determine the socio-demographic factors for the habitual use of paan, chaalia, gutka, niswar and tumbaku among adolescents and adults of an urban squatter settlement of Karachi.

## Results

A total of 425 subjects were enrolled. Punjabi [167(40.0%)], Pathan [113(27.0%)], Sindhi [67(16.0%)] and Mohajir [40(9.6%)] groups were the main ethnicities. Further analysis was conducted only among these ethnicities (387 study subjects). Males and females each constituted approximately 50.0% [193] of sample. The mean age of sample was 28.3 ± 12.9 (Mean ± SD) years. Adolescents (age 10–18 years) and adults (age > 18 years) formed 23.7% [102] and 76.3% [278], respectively. Fifty-nine percent [230] of the subjects were married (See Table-1).

**Table 1:** Distribution of the gender, age groups, marital status and ethnicities in a Pakistani squatter settlement*

	Number (N)	Gender		Age Group		Marital Status	
		
		Male	Female	Adolescents	Adults	Non-Married	Married
Punjabi	167	65	102	36	127	61	105
Pathan	113	60	52	31	81	50	63
Sindhi	67	39	28	18	47	31	36
Mohajir	40	29	11	5	35	14	26
Others	38	-	-	-	-	-	-

* Less number of subjects in groups are due to missing data

Table-2, 3 and 4 describe the distribution of daily chewing habits according to gender, age and marital status. Except chaalia, males were more likely to use all the studied substances on daily basis than females. Daily use of chaalia was more common among adolescents than adults. On the other hand, adults were more likely to use niswar on a daily basis than adolescents. Non-married subjects were more likely to use chaalia and gutka than married.

**Table 2:** Male gender as predictor of daily use of betel, areca and smokeless tobacco products in a Pakistani squatter settlement

	Prevalence (%)		Univariate OR [95% CI]
		
	Males	Females	
Paan	12.4	1.0	13.56 [3.16–58.22]
Chaalia	19.7	22.3	0.85 [0.52–1.40]
Gutka	13.5	0.5	29.69 [4.01–219.70]
Niswar	25.4	5.2	6.22 [3.05–12.70]
Tumbaku	13.5	1.0	14.86 [3.48–63.56]
Overall	50.3	28.5	2.53 [1.66–3.86]

**Table 3:** Adolescent age group as predictor of daily use of betel, areca and smokeless tobacco products in a Pakistani squatter settlement

	Prevalence (%)		Univariate OR [95% CI]
		
	Adolescents	Adults	
Paan	5.9	7.2	0.81 [0.31–2.07]
Chaalia	40.2	14.4	4.00 [2.38–6.72]
Gutka	9.8	6.1	1.67 [0.74–3.77]
Niswar	3.9	19.4	0.17 [0.06–0.48]
Tumbaku	4.9	8.3	0.57 [0.21–1.55]
Overall	45.1	37.8	1.35 [0.85–2.14]

**Table 4:** Marital status as predictor of daily use of betel, areca and smokeless tobacco products in a Pakistani squatter settlement

	Prevalence (%)		Univariate OR [95% CI]
		
	Non-Married	Married	
Paan	9.0	5.2	1.79 [0.81–3.98]
Chaalia	32.1	13.9	2.92 [1.77–4.82]
Gutka	12.8	3.0	4.68 [1.93–11.37]
Niswar	12.8	17.0	0.72 [0.40–1.29]
Tumbaku	7.7	7.0	1.11 [0.51–2.43]
Overall	48.7	33.5	1.88 [1.24–2.86]

Table-5 describes the relation of daily use of studied substances with ethnicity along with the mean frequency of use (Mean *freq*) among daily users. Mohajirs had significantly greater use of paan and tumbaku than other ethnicities. Mohajirs also had a greater use of gutka than Punjabi and Pathan ethnicities. Pathan ethnicity had a greater use of niswar than Sindhis. However, daily use of chaalia was not related to ethnicity.

**Table 5:** Ethnicity as predictor of daily use of betel, areca and smokeless tobacco products in a Pakistani squatter settlement

	Prevalence (%)	Univariate OR [95% CI]	Mean *Freq*
Paan			

Sindhi	6.0%	0.25 [0.07–0.91]	8.7
Punjabi	7.2%	0.31 [0.12–0.82]	6.5
Pathan	1.8%	0.07 [0.01–0.36]	11.0
Mohajir	20.2%	1.00	8.0

Chaalia			

Sindhi	25.4%	NS	4.8
Punjabi	17.4%	NS	6.7
Pathan	21.2%	NS	8.9
Mohajir	30.0%	1.00	5.9

Gutka			

Sindhi	6.0%	NS	5.0
Punjabi	6.6%	0.33 [0.12–0.92]	10.7
Pathan	4.4%	0.22 [0.06–0.73]	5.4
Mohajir	17.5%	1.00	7.4

Niswar			

Sindhi	6.0%	1.00	24.0
Punjabi	12.6%	NS	11.6
Pathan	24.8%	5.18 [1.73–15.53]	16.7
Mohajir	15.0%	NS	14.2

Tumbaku			

Sindhi	3.0%	0.12 [0.02–0.61]	4.5
Punjabi	7.8%	0.34 [0.13–0.88]	10.5
Pathan	4.4%	0.18 [0.06–0.61]	11.8
Mohajir	20.0%	1.00	8.1

Overall			

Sindhi	34.3%	NS	10.5
Punjabi	34.7%	NS	13.4
Pathan	46.0%	NS	15.2
Mohajir	50.0%	1.00	16.8

NS = Difference not statistically significant

## Discussion

The socio-economic and demographic profile of the sample represents a multi-ethnic, low socio-economic group of Pakistan [14]. As large section of population of Pakistan belongs to this group [14] and chewing habits are more commonly practiced by this disadvantaged segment of the society [5], therefore such a population makes an ideal choice for the study.

In our study sample, we observed a higher prevalence of all chewable products, except chaalia, among males. Daily use of chaalia was higher among adolescents while that of niswar was higher among adults. The prevalence of other chewable products was not different significantly among the two age groups. Overall daily use of chewable products, especially chaalia and gutka, was higher in the non-married group than in those who were married. Paan, gutka and tumbaku were associated with Mohajir ethnicity while niswar was associated with Pathan ethnicity.

High prevalence of chewing habits among different factions of Pakistani population is consistent with previous reports on this subject [2,3,6,13]. Moreover, our data has the advantage of being more extensive in terms of diversity of population and chewing substances studied. Males demonstrated a higher prevalence of all substances containing smokeless tobacco in varying amounts. This difference might be a reflection of higher prevalence of smoking tobacco among males [3]. Or it can be due to the social set up of the region, where males have social freedom and easy access to available chewable products. It was also commonly noted that females have more access to these products through their children and that may be the reason of similar high use of chaalia among both the children (adolescents) and females. The differential use of chewable products by gender is also reflected in the greater incidence of oral cavity-pharynx (M:F 30.7: 23.5) and laryngeal (M:F 8.5:1.5) cancers among males in this region (number represent age standardized incidence rates per 100,000) [10,15].

Higher prevalence of chaalia and gutka use among youth was demonstrated by this study. This reflects that industrially prepared chewable products containing betel, areca and tobacco together, marketed in bright, attractive sachets with appealing brand names like 'Sir', 'Shahi (royal)', are gaining popularity, especially among the younger population [1,9,16]. These eye-catching labels are commonly available at the tuck shops of most schools and communities of lower socio-economic strata [5]. Manufacturers promote these substances as being safe and socially acceptable. Advertisement is done by displaying media icons using these substances as symbolic gestures [16]. As an estimate in India, these industries are growing at an annual rate of 25–30% with a significant share of export to Pakistan [1,5,9,11].

A study done on school children of a Karachi population reported that more than 74% of them used chewable items on daily basis [13]. This tendency will have dire public health consequences as time trends analysis of oral cavity cancer in Karachi has already reported an up to 200% increase in cases among lower socio-economic strata between 1998 and 2002 [11]. A trend towards younger age at diagnosis was also observed. Presently, a third of cases occur among patients aged 40 years or younger [11].

Analysis of ethnic correlates of daily use of chewable products revealed that paan, gutka and tumbaku use were higher in Mohajir ethnicity. Similarly, niswar was more popular in Pathan ethnicity. These findings are consistent with local perceptions that immigrants from different parts of the country practice chewing habits as part of the culture they have brought from the area of their origin. For instance, in Sindh province of Pakistan, one finds the red spittle of paan on sideways more frequently than any other region. It was interesting to observe that for most substances, Pathan ethnicity had the highest mean frequency per day among daily users. This suggests that even if a smaller number of individuals consume a particular substance in Pathan ethnicity, those who use it have a very high frequency, putting them at particularly high risk of head and neck cancers.

While interpreting the results of this study following points should be kept in mind. The study did not take account of dependency of behavior where one male and a female were recruited from the same household. It is likely that the habit of one person will affect the habit of others in the same household. However, in approximately half of the sample, only one person (either a male or a female) could be recruited from a single household due to their unavailability. Therefore it may have little effect on these results, if any.

To discourage the consumption of chewing products of betel, areca and tobacco is the first step towards the control of epidemic of head and neck cancers in Pakistan [9] and requires radical measures involving health care professionals, media, policy makers and the community [16]. Unfortunately, insufficient data exists on the efficacy of various interventions to help develop guidelines [17]. Studies, including meta-analyses, have concluded that behavioral interventions are successful measures in reducing use of smokeless tobacco [18-20]. It is recommended that health care professionals should routinely assess and record smokeless tobacco use and rigorously inform the patients about its potential hazards [17]. Mass media campaign about the hazards of chewable tobacco has been shown to be effective in some studies [21-23]. Visual representation of the handicap caused by the use of these products may be effective for this purpose. Warning signs should accompany sachets and advertisements of these items, as used for cigarettes. Chaalia sponsored music videos showing TV stars using these products as a symbolic offering should be countered. Success of these tactics in smoking cessation (e.g. Fairness doctrine, Truth campaign, etc.) endows a promising aspect, although the efficacy remains to be established for cessation of smokeless tobacco products [24]. Health authorities have to play a pivotal role to formulate the policies to limit the industry, import, marketing and use of these items. However, as has been experienced, it requires strong political commitment [2].

These strategies may be more effective and less costly if they are tailored according to the preferred chewable substances used by a specific socio-economic group. For instance, awareness campaign in mohajirs in Karachi should be targeted against paan, gutka and tumbaku more specifically, while a similar programme in Pathan populated NWFP province should target niswar. Sale of industrially prepared chewable products should be banned in schools. Similarly, findings from our study can play an important role while planning targeted interventions in specific groups which are even at higher risk of this behavior. Specifically designed distinct prevention and cessation programmes for different risk groups have also been suggested by Pitney *et al *[25]. Nevertheless, importance of general interventions targeted against smoking and chewable products cannot be overestimated. Without such measures, the incidence of head and neck cancers will not only keep on rising, but also keep on affecting younger age groups.

## Conclusion

Prevalence of use of chewable products is high in Pakistan with particularly high use of certain substances related with socio-demographic profiles. Industrially prepared products, chaalia and gutka, are gaining popularity among the youth. Policies and focused interventions can be developed taking into consideration the preferred use of products among different socio-demographic groups.

## Methods

This cross-sectional, house to house survey was conducted by a group of medical students among the adolescents and adults (≥10 years age) of Bilal Colony, Karachi. Sample size was calculated to detect an association at 80% power and 95% confidence level, assuming the prevalence of chewing habits to be 2.5 times higher among males than females.

The study was conducted in compliance with 'Ethical principles for medical research involving human subjects' of Helsinki Declaration [26]. Study protocol was discussed among the students and facilitating faculty for possible ethical concerns. Verbal informed consent was obtained from the subjects.

Every third house was selected systematically after random selection of the first household. From every household, one male and one female (age ≥ 10 years) was recruited, at the most. If only one of the male or female was present, he or she was recruited in the study. Random selection was made if more than one eligible person was present. The survey was conducted between 1000 and 1500 hours.

The information was collected on a standard, pre-tested structured questionnaire determining the frequency of use. Apart from demographic information, subjects were inquired about their chewing practices of paan, chaalia, gutka, niswar and tumbaku separately. The subjects were classified into 'daily' (average use of once a day or more), and 'not daily' users (average use of less than once a day).

Data was entered and analyzed in Statistical Package for Social Sciences 13.0 (SPSS 13.0). Descriptive statistics of socio-demographic information and practices of each of chewing products were determined. Univariate Odds Ratios with 95% confidence intervals and p-values were determined using chi-square to look at the association between socio-demographic variables and practices of chewing products. For all purposes, a p-value of <0.05 was considered as the criteria of significance.

## Conflict of interest

The author(s) declare that they have no competing interest.

## Authors' contributions

This study was carried out by a group of medical students (SM, RM, MM, KAM, FM, AM and MRK) as an essential part of their rotation in Community Health Sciences. MRK and SG conceived the idea. ZF, SM and KAM composed design and protocol. SM, RM, MM, KAM, FM, AM and MRK administered questionnaires. MM, RM, FM and AM managed, analyzed and interpreted the data. MRK, ZF and SG wrote the manuscript. ZF and MRK did overall supervision. All authors approved the final manuscript.
